# Supplementary material for: Observation of bulk quadrupole in topological heat transport
Source: Nat Commun. 2023 Jun 5;14:3252. doi: 10.1038/s41467-023-39117-w (PMC10241824; doi:10.1038/s41467-023-39117-w)
Supplement: Supplementary file 1 — Supplementary Information [file 41467_2023_39117_MOESM1_ESM.pdf]

Supplementary Information of "Observation of Bulk Quadrupole in Topological Heat Transport"

Guoqiang Xu[1#], Xue Zhou[2#], Shuihua Yang[1#], Jing Wu[3, 4], and Cheng-Wei Qiu[1*]

[1]Department of Electrical and Computer Engineering, National University of Singapore, Kent Ridge 117583, Republic of Singapore

[2]School of Computer Science and Information Engineering, Chongqing Technology and Business University, Chongqing, 400067, China

[3]Institute of Materials Research and Engineering, Agency for Science, Technology and Research, Singapore, Singapore

[4]Department of Materials Science and Engineering, National University of Singapore, Singapore, Singapore

[#]These authors contributed equally.

[*]Correspondence to: chengwei.qiu@nus.edu.sg

Supplementary Note 1. Effective square lattice for the quadrupole topological phases in heat transport

We first start with the heat exchanges and corresponding heat transport processes of one site described by Eq. (1) of the main content. The right terms of Eq. (1) respectively indicate the conductive and convective processes, as well as the total heat exchanges between the target and its neighbors. Since the heat exchanges are induced by the convective processes, the total exchanged energies can be described by Newton's cooling law, i.e., $\sum Q_{intracell/intercell} = \sum h_{intracell/intercell} \cdot \Delta T$, where h denotes the convective heat transfer coefficient. For the current model, each target site ($T_{i,j}$, light-red dashed border) connects four neighbors in the system (Fig. S1a), indicating four independent heat exchange terms related to the target site. Among these heat exchanges, two of them describe the intracell couplings between two neighboring sites within one four-site unit-structure (orange lines of Supplementary Figure 1**a**), while another two represent the intercell couplings of the heat exchanges between the neighboring sites of two adjacent unit structures (black lines of Supplementary Figure 1**a**). Due to the different heat exchange capabilities of the intracell and intercell channels, we make $\sum Q_{intracell} = \beta \cdot \sum Q_{intercell}$, which can be realized by engineering their convective heat transfer coefficient via modulating the total heat exchange areas of corresponding channels based on Newton's cooling law.

A 2D effective square lattice can be created with the proposed unit-structure, which possesses two advective components respectively along x and y directions. Such a 2D thermal lattice further leads to a thermal grid system (Supplementary Figure 1**a**), and its energy equation can be expressed as

$$\frac{\partial T_{ij}}{\partial t} = \sum_{i}^{4}\left(\frac{\kappa}{\rho c}\frac{\partial^2 T_{ij}}{\partial \mathbf{x}^2} + \frac{\partial\left(\Omega_i \cdot R(\mathbf{x}) \cdot T_{ij}\right)}{\partial \mathbf{x}}\right) + \sum_{j}^{4}\left(\frac{\kappa}{\rho c}\frac{\partial^2 T_{ij}}{\partial \mathbf{y}^2} + \frac{\partial\left(\Omega_j \cdot R(\mathbf{y}) \cdot T_{ij}\right)}{\partial \mathbf{y}}\right). \tag{1}$$

where $\Omega_i = \Omega_{I/II}\cos(\theta)$ and $\Omega_j = \Omega_{I/II}\sin(\theta)$. $R(\mathbf{x})$ and $R(\mathbf{y})$ denote the directional components of one site. i and j depict the spatial information of each site. For simplification, we make the thermal lattice follow the spatial periodicity of $4a$ along the x and y directions, where a is the distance between the centers of neighboring sites (Fig. 1**b** of the main content). That is, the effective lattice constants of the two directions are both $4a$ (Supplementary Figure 1**b**), and the advections follow the relations: $\Omega_{i+4}(\mathbf{x}) = \Omega_i(\mathbf{x} + 4a)$ and $\Omega_{j+4}(\mathbf{y}) = \Omega_j(\mathbf{y} + 4a)$. Taking into account these advective

periodicities and the wave-like temperature propagation, Supplementary Equation 1 can be further written as a similar form of Schrodinger's equation

$$\left(\frac{\kappa}{\rho c}\nabla^2+\nabla\left(\Omega_{ij}\cdot R(\mathbf{x},\mathbf{y})\right)\right)\cdot\psi(\mathbf{x},\mathbf{y})=E\psi(\mathbf{x},\mathbf{y}). \quad (2)$$

Due to the translation symmetry of the advective configurations, the gradients of decoupled velocity fields along the two vectors (**x**) and (**y**) further result in effective potential fields. Then, Supplementary Equation 2 can be solved based on Bloch theorem: $\psi(\mathbf{x}+4a,\mathbf{y}+4a)=e^{ik_{t,x}\cdot 4a+ik_{t,y}\cdot 4a}\psi(\mathbf{x},\mathbf{y})$. Here, $k_{t,x}$ and $k_{t,y}$ denote the effective Bloch wave numbers along the two vectors. Considering the continuous boundary conditions at the interface of neighboring sites, we can indicate the following relations based on Newton cooling law

$$\begin{aligned}
&\text{Along } x \text{ direction: }\begin{cases}
T_{ij}(\mathbf{x})=T_{i+1j}(\mathbf{x}),\\
q_{ij}(\mathbf{x})=q_{i+1j}(\mathbf{x}),\\
q_{i-3j\sim ij}(\mathbf{x})=\sum\left(-\kappa\left(\nabla T_{i-3j\sim ij}(\mathbf{x})\right)+\omega_{i-3j\sim ij}R(\mathbf{x})T_{i-3j\sim ij}(\mathbf{x})\right)\\
\quad=\sum h_{i-3j\sim ij}\left(T_{i-2j\sim i+1j}-T_{i-3j\sim ij}\right)=h\left(T_{i+1j}-T_{i-3j}\right).
\end{cases}\\
&\text{Along } y \text{ direction: }\begin{cases}
T_{ij}(\mathbf{y})=T_{ij+1}(\mathbf{y}),\\
q_{ij}(\mathbf{y})=q_{ij+1}(\mathbf{y}),\\
q_{ij-1\sim ij+2}(\mathbf{y})=\sum\left(-\kappa\left(\nabla T_{ij-1\sim ij+2}(\mathbf{y})\right)+\omega_{ij-1\sim ij+2}R(\mathbf{y})T_{ij-1\sim ij+2}(\mathbf{y})\right)\\
\quad=\sum h_{ij-1\sim ij+2}\left(T_{ij\sim ij+3}-T_{ij-1\sim ij+2}\right)=h\left(T_{ij+3}-T_{ij-1}\right).
\end{cases}
\end{aligned} \quad (3)$$

In Supplementary Equation 3, the continuous boundaries ensure the accordance of temperature fields and heat fluxes of the neighboring sites at their interfaces. $q_{i\text{-}3j\sim ij}$ (**x**) and $q_{ij\text{-}1\sim ij+2}$ (**y**) denote the heat flux components along the two lattice vectors. Considering the periodic potentials of the eigenvectors, the heat flux components should satisfy the following relation: $q_{ij\sim i+1j}(\mathbf{x})=h\left(T_{i+1j}(\mathbf{x})-T_{ij}(\mathbf{x})\right)=\beta e^{ik_{t,x}\cdot 4a}q_{i-1j\sim ij}(\mathbf{x})$ and $q_{ij+2\sim ij+3}(\mathbf{y})=h\left(T_{ij+3}(\mathbf{y})-T_{ij+2}(\mathbf{y})\right)=\beta e^{ik_{t,y}\cdot 4a}q_{ij+1\sim ij+2}(\mathbf{y})$. Taking these heat flux components into the governing function (Eq. (1) of the main content), the thermal couplings at the interfaces of two neighboring lattices can be regarded as effective hopping.

For the cases induced by the Hermitian advection ($\Omega_I\neq\Omega_{II},\beta=1$), the heat flux components should be same between any two neighboring sites/lattices, i.e., $\frac{q_{i-3j\sim ij}(\mathbf{x})}{4a}=\frac{q_{i+1j\sim i+4j}(\mathbf{x})}{4a}=\frac{q_{ij\sim i+1j}(\mathbf{x})}{a}=\frac{q_{i+1j\sim i+2j}(\mathbf{x})}{a}$, and $\frac{q_{ij-1\sim ij+2}(\mathbf{y})}{4a}=$

$\frac{q_{ij+3\sim ij+6}(\mathbf{y})}{4a} = \frac{q_{ij+2\sim ij+3}(\mathbf{y})}{a} = \frac{q_{ij+1\sim ij+2}(\mathbf{y})}{a}$. In that case, the effective Bloch wave numbers $k_{t,x}$ and $k_{t,y}$ are dependent on the imposed advections, since the periodic wave properties and effective oscillations of the system are determined by their values. Considering the neighboring sites at the interfaces of neighboring lattices (Fig. 1**a**), the continuous temperature condition leads to the following relations: 1) $T_{ij}(\mathbf{x}) = e^{i(k_x x_{ij} - \Omega_{II}\cos(\theta)\cdot t + \varphi_x)}$, $T_{i+1j}(\mathbf{x}) = e^{i(k_x x_{i+1j} - \Omega_I \cos(\theta)\cdot t + \varphi_x)} = e^{i(k_{t,x}\cdot 4a)} T_{ij}(\mathbf{x})$, and 2) $T_{ij+2}(\mathbf{y}) = e^{i(k_y y_{ij+2} - \Omega_{II}\sin(\theta)\cdot t + \varphi_y)}$, $T_{ij+3}(\mathbf{y}) = e^{i(k_y y_{ij+3} - \Omega_I \sin(\theta)\cdot t + \varphi_y)} = e^{i(k_{t,y}\cdot 4a)} T_{ij+2}(\mathbf{y})$. Then, the effective Bloch wave numbers for the cases induced by Hermitian advection (Figs. 1 and 2) can be expressed as: $k_{t,x} = \frac{(\Omega_I - \Omega_{II})\cos(\theta)\cdot t}{4a}$, and $k_{t,y} = \frac{(\Omega_I - \Omega_{II})\sin(\theta)\cdot t}{4a}$.

For the cases induced by the non-Hermitian thermal couplings ($\Omega_I = \Omega_{II}, \beta \neq 1$), the heat flux between neighboring lattices should be same. Moreover, the intercell and intracell heat flux should simultaneously satisfy the relations of specific β at one site, i.e., $\frac{q_{i-3j\sim ij}(\mathbf{x})}{4a} = \frac{q_{i+1j\sim i+4j}(\mathbf{x})}{4a}, \frac{q_{ij\sim i+1j}(\mathbf{x})}{a} = \beta \frac{q_{i-1j\sim ij}(\mathbf{x})}{a}$, and $\frac{q_{ij-1\sim ij+2}(\mathbf{y})}{4a} = \frac{q_{ij+3\sim ij+6}(\mathbf{y})}{4a}, \frac{q_{ij+2\sim ij+3}(\mathbf{y})}{a} = \beta \frac{q_{ij+1\sim ij+2}(\mathbf{y})}{a}$. In that case, the effective Bloch wave numbers $k_{t,x}$ and $k_{t,y}$ are dependent on the difference between the convective heat transfer coefficients of the intercell and intracell channels. Considering the interfaces between two neighboring lattices (Fig. 3**a**), the following conditions along the two vectors are indispensable: 1) $h_{intercell}\left(T_{i+1j}(\mathbf{x}) - T_{ij}(\mathbf{x})\right) = h_{intracell}\left(T_{ij}(\mathbf{x}) - T_{i-1j}(\mathbf{x})\right)$, $T_{i+1j}(\mathbf{x}) = e^{i(k_x x_{i+1j} - \Omega_I \cos(\theta)\cdot t + \varphi_x)} = e^{i(k_{t,x}\cdot 4a)} T_{ij}(\mathbf{x})$, and $T_{i-1j}(\mathbf{x}) = e^{-i(k_x\cdot a)} T_{ij}(\mathbf{x})$; and 2) $h_{intercell}\left(T_{ij+3}(\mathbf{y}) - T_{ij+2}(\mathbf{y})\right) = h_{intracell}\left(T_{ij+2}(\mathbf{x}) - T_{ij+1}(\mathbf{y})\right)$, $T_{ij+3}(\mathbf{y}) = e^{i(k_y y_{ij+3} - \Omega_I \sin(\theta)\cdot t + \varphi_y)} = e^{i(k_{t,y}\cdot 4a)} T_{ij+2}(\mathbf{y})$, and $T_{ij+1}(\mathbf{x}) = e^{-i(k_y\cdot a)} T_{ij+2}(\mathbf{x})$. Then, the effective Bloch wave numbers for the cases induced by non-Hermitian thermal couplings (Figs. 3 and 4) can be expressed as: $k_{t,x} = Re\left(\frac{-i\cdot ln(\beta(1-e^{-i(k_x\cdot a)})+1)}{4a}\right)$, and $k_{t,y} = Re\left(\frac{-i\cdot ln\left(\beta\left(1-e^{-i(k_y\cdot a)}\right)+1\right)}{4a}\right)$.

Supplementary Note 2. Theoretical model for thermal quadrupole topological phases

The thermal process of each unit-structure (Figs. 1**b** and **3b**) can be expressed as Supplementary Equation 4.

$$\rho c\frac{\partial T_{i,j}}{\partial t}=\kappa\left(\nabla_x^2 T_{i,j}+\nabla_y^2 T_{i,j}\right)\pm\rho c\left(\Omega_I R_x\cos\theta\right)\nabla_x T_{i,j}\pm\rho c\left(\Omega_I R_y\sin\theta\right)\nabla_y T_{i,j}$$
$$\underbrace{-\frac{h_x}{a_x}\left(T_{i,j}-T_{i-1,j}\right)+\frac{h_y}{a_y}\left(T_{i,j}-T_{i,j-1}\right)}_{\text{intracell}}\underbrace{-\frac{\beta h_x}{a_x}\left(T_{i+1,j}-T_{i,j}\right)+\frac{\beta h_y}{a_y}\left(T_{i,j+1}-T_{i,j}\right)}_{\text{intercell}}.$$
$$\rho c\frac{\partial T_{i,j-1}}{\partial t}=\kappa\left(\nabla_x^2 T_{i,j-1}+\nabla_y^2 T_{i,j-1}\right)\mp\rho c\left(\Omega_{II} R_x\cos\theta\right)\nabla_x T_{i,j-1}\mp\rho c\left(\Omega_{II} R_y\sin\theta\right)\nabla_y T_{i,j-1}$$
$$\underbrace{+\frac{h_x}{a_x}\left(T_{i-1,j-1}-T_{i,j-1}\right)+\frac{h_y}{a_y}\left(T_{i,j}-T_{i,j-1}\right)}_{\text{intracell}}\underbrace{+\frac{\beta h_x}{a_x}\left(T_{i+1,j-1}-T_{i,j-1}\right)+\frac{\beta h_y}{a_y}\left(T_{i,j-1}-T_{i,j-2}\right)}_{\text{intercell}}.$$
$$\rho c\frac{\partial T_{i-1,j-1}}{\partial t}=\kappa\left(\nabla_x^2 T_{i-1,j-1}+\nabla_y^2 T_{i-1,j-1}\right)\pm\rho c\left(\Omega_I R_x\cos\theta\right)\nabla_x T_{i-1,j-1}\pm\rho c\left(\Omega_I R_y\sin\theta\right)\nabla_y T_{i-1,j-1}\tag{4}$$
$$\underbrace{+\frac{h_x}{a_x}\left(T_{i,j-1}-T_{i-1,j-1}\right)+\frac{h_y}{a_y}\left(T_{i-1,j}-T_{i-1,j-1}\right)}_{\text{intracell}}\underbrace{+\frac{\beta h_x}{a_x}\left(T_{i-1,j-1}-T_{i-2,j-1}\right)+\frac{\beta h_y}{a_y}\left(T_{i-1,j-1}-T_{i-1,j-2}\right)}_{\text{intercell}}.$$
$$\rho c\frac{\partial T_{i-1,j}}{\partial t}=\kappa\left(\nabla_x^2 T_{i-1,j}+\nabla_y^2 T_{i-1,j}\right)\mp\rho c\left(\Omega_{II} R_x\cos\theta\right)\nabla_x T_{i-1,j}\mp\rho c\left(\Omega_{II} R_y\sin\theta\right)\nabla_y T_{i-1,j}$$
$$\underbrace{-\frac{h_x}{a_x}\left(T_{i,j,n}-T_{i-1,j}\right)+\frac{h_y}{a_y}\left(T_{i-1,j}-T_{i-1,j-1}\right)}_{\text{intracell}}\underbrace{-\frac{\beta h_x}{a_x}\left(T_{i-1,j}-T_{i-2,j}\right)+\frac{\beta h_y}{a_y}\left(T_{i-1,j+1}-T_{i-1,j}\right)}_{\text{intercell}}.$$

Then, the effective Hamiltonian of a four-site unit-structure can now be written as

$$H=i\cdot\begin{bmatrix} i\cdot\Omega_I\left(\cos\theta+\sin\theta\right) & Q_c\left(1+\beta e^{ik_{t,x}\cdot a}\right) & 0 & Q_c\left(1+\beta e^{-ik_{t,y}\cdot a}\right)\\ Q_c\left(1+\beta e^{-ik_{t,x}\cdot a}\right) & -i\cdot\Omega_{II}\left(\cos\theta+\sin\theta\right) & Q_c\left(1+\beta e^{-ik_{t,y}\cdot a}\right) & 0\\ 0 & Q_c\left(1+\beta e^{ik_{t,y}\cdot a}\right) & -i\cdot\Omega_I\left(\cos\theta+\sin\theta\right) & Q_c\left(1+\beta e^{-ik_{t,x}\cdot a}\right)\\ Q_c\left(1+\beta e^{ik_{t,y}\cdot a}\right) & 0 & Q_c\left(1+\beta e^{ik_{t,x}\cdot a}\right) & i\cdot\Omega_{II}\left(\cos\theta+\sin\theta\right)\end{bmatrix}$$
$$-i\cdot\left(\frac{\kappa}{\rho c}\cdot\left(k_x^2+k_y^2\right)+\left(1+\beta\right)Q_c\right)\cdot I_{4\times4}.\tag{5}$$

The terms $Q_c(1+\beta e^{\pm ik_{t,x}a})$ and $Q_c\left(1+\beta e^{\pm ik_{t,y}a}\right)$ denote the effective hopping in imaginary parts, which originate from the dissipative thermal couplings between neighboring sites. The terms $\pm\Omega_I(\cos\theta+\sin\theta)$ and $\pm\Omega_{II}(\cos\theta+\sin\theta)$ induced by the imposed advections act as the on-site energies in real parts. The intercell couplings should follow the Bloch theorem, where $k_{t,x}$ and $k_{t,y}$ are the directional components of the effective Bloch wave number k_t. Solving the eigenvalue problem with the wave-like temperatures, the eigenvalues can be obtained

$$E=i\cdot\left(\frac{\kappa}{\rho c}\cdot\left(k_x^2+k_y^2\right)+\frac{(1+\beta)h}{\rho ca}\right)+\frac{1}{2}\left(\Omega_I+\Omega_{II}\right)\left(\cos\theta+\sin\theta\right)$$
$$\pm\frac{1}{2}\sqrt{\begin{matrix}\left(\Omega_I-\Omega_{II}\right)^2\left(\cos\theta+\sin\theta\right)^2-8Q_c^2\left(\cos\left(k_{t,x}a\right)+\cos\left(k_{t,y}a\right)+1+\beta\right)\\ \pm4Q_c\sqrt{\left(2\beta\cos\left(k_{t,y}a\right)+\beta^2+1\right)\left(4Q_c^2\left(2\beta\cos\left(k_{t,x}a\right)+\beta^2+1\right)-\left(\Omega_I-\Omega_{II}\right)^2\left(\cos\theta+\sin\theta\right)^2\right)}\end{matrix}}\tag{6}$$

Considering the above eigenvalues, a band gap $\Delta = E_+ - E_- = \sqrt{\begin{matrix} (\Omega_I - \Omega_{II})^2(\cos\theta + \sin\theta)^2 \\ -8(Q_c)^2\left(\cos(k_{t,x}a) + \cos(k_{t,x}a) + 1 + \beta\right) \\ \pm 4Q_c\sqrt{\left(2\beta\cos(k_{t,y}a) + 1 + \beta^2\right)\left(4\left(\frac{h}{\rho c a}\right)^2\left(2\beta\cos(k_{t,x}a) + 1 + \beta^2\right) - (\Omega_I - \Omega_{II})^2(\cos\theta + \sin\theta)^2\right)} \end{matrix}}$ can be observed. Note that both the advections (Ω_I and Ω_{II}) and the thermal coupling strength ratio (β) determine the band structure. This suggests the potential of revealing quadrupole topological phases either in an independent real/imaginary band or a complex band. The minimum band gap is $\Delta = \sqrt{(\Omega_I - \Omega_{II})^2 - 8(Q_c)^2}$ under the condition of $\beta = 1$. When the advections follow the relation of $|\Delta\Omega| = |\Omega_I - \Omega_{II}| \geq 2\sqrt{2}Q_c$, the system would be always gapped ($\Delta > 0$) with the same intercell and intracell thermal coupling strengths. Then, the eigenvectors without considering the global shifting-terms can be also observed

$$|\psi_1\rangle = i\cdot\begin{pmatrix} \dfrac{m_\Omega\left(h^2\left(m_x+m_y\right)+\sqrt{h^4 m_x m_y-\frac{h^2 m_y m_\Omega^2}{4}}\left(\sqrt{8\sqrt{h^2 m_y\left(h^2 m_x-\frac{m_\Omega^2}{4}\right)}-8h^2 m_c+m_\Omega^2}+1\right)\right)\left(\beta e^{-i\frac{k_{t,y}a}{2}}+1\right)}{2h\left(\frac{m_y m_\Omega}{4}\sqrt{8\sqrt{h^2 m_y\left(h^2 m_x-\frac{m_\Omega^2}{4}\right)}-8h^2 m_c+m_\Omega^2}+2m_c\sqrt{h^2 m_x m_y-\frac{m_y m_\Omega^2}{4}}-\left(2h^2 m_x m_y-\frac{m_y m_\Omega^2}{4}\right)\right)} \\ \dfrac{h^2\sqrt{8\sqrt{h^2 m_y\left(h^2 m_x-\frac{m_\Omega^2}{4}\right)}-8h^2 m_c+m_\Omega^2}\cdot\left(\left(-\beta^2 e^{-i\frac{k_{t,x}a}{2}}-\beta\right)\cos\left(k_{t,y}a\right)+\left(i\beta^2\sin\left(k_{t,y}a\right)-\beta\right)\cos\left(k_{t,x}a\right)+\left(\beta^2\sin\left(k_{t,y}a\right)+i\beta\right)\sin\left(k_{t,x}a\right)+i\beta\sin\left(k_{t,y}a\right)-1\right)}{\sqrt{8\sqrt{h^2 m_y\left(h^2 m_x-\frac{m_\Omega^2}{4}\right)}-8h^2 m_c+m_\Omega^2}\sqrt{h^4 m_x m_y-\frac{h^2 m_y m_\Omega^2}{4}}-m_\Omega\left(\sqrt{h^4 m_x m_y-\frac{h^2 m_y m_\Omega^2}{4}}+h^2 m_y\right)} \\ \dfrac{2h\left(\beta e^{-i\frac{k_{t,x}a}{2}}+1\right)\left(-\sqrt{h^2 m_y\left(h^2 m_x-\frac{m_\Omega^2}{4}\right)}+h^2 m_y\right)}{-\sqrt{8\sqrt{h^2 m_y\left(h^2 m_x-\frac{m_\Omega^2}{4}\right)}-8h^2 m_c+m_\Omega^2}\sqrt{h^4 m_x m_y-\frac{h^2 m_y m_\Omega^2}{4}}+m_\Omega\left(-\sqrt{h^4 m_x m_y-\frac{h^2 m_y m_\Omega^2}{4}}+h^2 m_y\right)} \\ 1 \end{pmatrix}. \quad (7)$$

$$
\left|\psi_2\right\rangle = i\cdot\begin{pmatrix}
\dfrac{m_\Omega\left(h^2\left(-m_x+m_y\right)+\sqrt{h^4 m_x m_y-\dfrac{h^2 m_y m_\Omega^2}{4}}\left(\sqrt{8\sqrt{h^2 m_y\left(h^2 m_x-\dfrac{m_\Omega^2}{4}\right)}-8h^2 m_c+m_\Omega^2}-1\right)\right)\left(\beta e^{-i\frac{k_{t,y}a}{2}}+1\right)}{2h\left(\dfrac{m_y m_\Omega}{4}\sqrt{8\sqrt{h^2 m_y\left(h^2 m_x-\dfrac{m_\Omega^2}{4}\right)}-8h^2 m_c+m_\Omega^2}+2m_c\sqrt{h^2 m_x m_y-\dfrac{m_y m_\Omega^2}{4}}-\left(2h^2 m_x m_y-\dfrac{m_y m_\Omega^2}{4}\right)\right)} \\
\dfrac{h^2\sqrt{8\sqrt{h^2 m_y\left(h^2 m_x-\dfrac{m_\omega^2}{4}\right)}-8h^2 m_c+m_\Omega^2}\cdot\left(\begin{array}{l}\left(-\beta^2 e^{-i\frac{k_{t,x}a}{2}}-\beta\right)\cos\left(k_{t,y}a\right)+\left(i\beta^2\sin\left(k_{t,y}a\right)-\beta\right)\cos\left(k_{t,x}a\right)\\+\left(\beta^2\sin\left(k_{t,y}a\right)+i\beta\right)\sin\left(k_{t,x}a\right)+i\beta\sin\left(k_{t,y}a\right)-1\end{array}\right)}{\sqrt{8\sqrt{h^2 m_y\left(h^2 m_x-\dfrac{m_\Omega^2}{4}\right)}-8h^2 m_c+m_\Omega^2}\sqrt{h^4 m_x m_y-\dfrac{h^2 m_y m_\Omega^2}{4}}+m_\Omega\left(\sqrt{h^4 m_x m_y-\dfrac{h^2 m_y m_\Omega^2}{4}}+h^2 m_y\right)} \\
\dfrac{-2h\left(\beta e^{-i\frac{k_{t,x}a}{2}}+1\right)\left(-\sqrt{h^2 m_y\left(h^2 m_x-\dfrac{m_\omega^2}{4}\right)}+h^2 m_y\right)}{\sqrt{8\sqrt{h^2 m_y\left(h^2 m_x-\dfrac{m_\Omega^2}{4}\right)}-8h^2 m_c+m_\Omega^2}\sqrt{h^4 m_x m_y-\dfrac{h^2 m_y m_\Omega^2}{4}}+m_\Omega\left(-\sqrt{h^4 m_x m_y-\dfrac{h^2 m_y m_\Omega^2}{4}}+h^2 m_y\right)} \\
1
\end{pmatrix}. \tag{8}
$$

$$
\left|\psi_3\right\rangle = i\cdot\begin{pmatrix}
\dfrac{m_\Omega\left(h^2\left(m_x+m_y\right)+\sqrt{h^4 m_x m_y-\dfrac{h^2 m_y m_\Omega^2}{4}}\left(\sqrt{-8\sqrt{h^2 m_y\left(h^2 m_x-\dfrac{m_\Omega^2}{4}\right)}-8h^2 m_c+m_\Omega^2}+1\right)\right)\left(\beta e^{-i\frac{k_{t,y}a}{2}}+1\right)}{2h\left(-\dfrac{m_y m_\Omega}{4}\sqrt{-8\sqrt{h^2 m_y\left(h^2 m_x-\dfrac{m_\Omega^2}{4}\right)}-8h^2 m_c+m_\Omega^2}+2m_c\sqrt{h^2 m_x m_y-\dfrac{m_y m_\Omega^2}{4}}+\left(2h^2 m_x m_y-\dfrac{m_y m_\Omega^2}{4}\right)\right)} \\
\dfrac{-h^2\sqrt{-8\sqrt{h^2 m_y\left(h^2 m_x-\dfrac{m_\Omega^2}{4}\right)}-8h^2 m_c+m_\Omega^2}\cdot\left(\begin{array}{l}\left(-\beta^2 e^{-i\frac{k_{t,x}a}{2}}-\beta\right)\cos\left(k_{t,y}a\right)+\left(i\beta^2\sin\left(k_{t,y}a\right)-\beta\right)\cos\left(k_{t,x}a\right)\\+\left(\beta^2\sin\left(k_{t,y}a\right)+i\beta\right)\sin\left(k_{t,x}a\right)+i\beta\sin\left(k_{t,y}a\right)-1\end{array}\right)}{\sqrt{-8\sqrt{h^2 m_y\left(h^2 m_x-\dfrac{m_\Omega^2}{4}\right)}-8h^2 m_c+m_\Omega^2}\sqrt{h^4 m_x m_y-\dfrac{h^2 m_y m_\Omega^2}{4}}+m_\Omega\left(\sqrt{h^4 m_x m_y-\dfrac{h^2 m_y m_\Omega^2}{4}}+h^2 m_y\right)} \\
\dfrac{2h\left(\beta e^{-i\frac{k_{t,x}a}{2}}+1\right)\left(\sqrt{h^2 m_y\left(h^2 m_x-\dfrac{m_\Omega^2}{4}\right)}+h^2 m_y\right)}{\sqrt{-8\sqrt{h^2 m_y\left(h^2 m_x-\dfrac{m_\Omega^2}{4}\right)}-8h^2 m_c+m_\Omega^2}\sqrt{h^4 m_x m_y-\dfrac{h^2 m_y m_\Omega^2}{4}}+m_\Omega\left(\sqrt{h^4 m_x m_y-\dfrac{h^2 m_y m_\Omega^2}{4}}+h^2 m_y\right)} \\
1
\end{pmatrix}. \tag{9}
$$

$$
\left|\psi_4\right\rangle = i\cdot\begin{pmatrix}
\dfrac{m_\Omega\left(h^2\left(m_x+m_y\right)+\sqrt{h^4m_xm_y-\dfrac{h^2m_ym_\Omega^2}{4}}\left(\sqrt{-8\sqrt{h^2m_y\left(h^2m_x-\dfrac{m_\Omega^2}{4}\right)}-8h^2m_c+m_\Omega^2}+1\right)\right)\left(\beta e^{-i\frac{k_{t,y}a}{2}}+1\right)}{2h\left(-\dfrac{m_ym_\Omega}{4}\sqrt{-8\sqrt{h^2m_y\left(h^2m_x-\dfrac{m_\Omega^2}{4}\right)}-8h^2m_c+m_\Omega^2}+2m_c\sqrt{h^2m_xm_y-\dfrac{m_ym_\Omega^2}{4}}+\left(2h^2m_xm_y-\dfrac{m_ym_\Omega^2}{4}\right)\right)} \\
\dfrac{h^2\sqrt{-8\sqrt{h^2m_y\left(h^2m_x-\dfrac{m_\Omega^2}{4}\right)}-8h^2m_c+m_\Omega^2}\cdot\left(\begin{array}{l}\left(-\beta^2e^{-i\frac{k_{t,x}a}{2}}-\beta\right)\cos\left(k_{t,y}a\right)+\left(i\beta^2\sin\left(k_{t,y}a\right)-\beta\right)\cos\left(k_{t,x}a\right)\\+\left(\beta^2\sin\left(k_{t,y}a\right)+i\beta\right)\sin\left(k_{t,x}a\right)+i\beta\sin\left(k_{t,y}a\right)-1\end{array}\right)}{-\sqrt{-8\sqrt{h^2m_y\left(h^2m_x-\dfrac{m_\Omega^2}{4}\right)}-8h^2m_c+m_\Omega^2}\sqrt{h^4m_xm_y-\dfrac{h^2m_ym_\Omega^2}{4}}+m_\Omega\left(\sqrt{h^4m_xm_y-\dfrac{h^2m_ym_\Omega^2}{4}}+h^2m_y\right)} \\
\dfrac{2h\left(\beta e^{-i\frac{k_{t,x}a}{2}}+1\right)\left(\sqrt{h^2m_y\left(h^2m_x-\dfrac{m_\Omega^2}{4}\right)}+h^2m_y\right)}{-\sqrt{-8\sqrt{h^2m_y\left(h^2m_x-\dfrac{m_\Omega^2}{4}\right)}-8h^2m_c+m_\Omega^2}\sqrt{h^4m_xm_y-\dfrac{h^2m_ym_\Omega^2}{4}}+m_\Omega\left(\sqrt{h^4m_xm_y-\dfrac{h^2m_ym_\Omega^2}{4}}+h^2m_y\right)} \\
1
\end{pmatrix}. \quad (10)
$$

In Supplementary Equations 7 ~ 10, $m_x=\beta^2+1+2\beta cos\left(k_{t,x}\cdot a\right)$, $m_y=\beta^2+1+2\beta cos\left(k_{t,y}\cdot a\right)$, $m_c=\beta^2+1+\beta\left(\cos\left(k_{t,x}\cdot a\right)+\cos\left(k_{t,y}\cdot a\right)\right)$, and $m_\Omega=\left(\Omega_I+\Omega_{II}\right)\left(\cos(\theta)+\sin(\theta)\right)$.

Supplementary Note 3. Effective quadrupole and over-coupling/under-coupling in heat transport

As a typical quadrupole in electrostatics, the simplest model consists of alternating positive and negative charges, arranged on the four corners of a square. Such a configuration leads to the zero monopole and dipole moments and the nonzero quadrupole moment. The electric potential can be expressed as $\varphi(\mathbf{R})=\frac{1}{4\pi\epsilon_0}\frac{1}{|\mathbf{R}|^3}\sum\frac{1}{2}Q_{ij}\widehat{R}_i\widehat{R}_j$, where ϵ_0 is the electric permittivity, and Q_{ij} denotes the components of the quadrupole moment tensor. Here, we recall the functions of charge density $\nabla(-\varepsilon\nabla\varphi)=\rho_{ele}$ in electrostatics and the persistent power density $\nabla(-\kappa\nabla T)=\rho_{heat}$ in thermal conduction. The two governing functions share the same form of Poisson equation, thus resulting in the similarity in describing the field distributions with the following equivalent parameters, i.e., $\varepsilon\rightarrow\kappa, \varphi\rightarrow T, \rho_{ele}\rightarrow\rho_{heat}$. In this case, the temperature field could also exhibit similar distributions to the electric potential $\varphi(\mathbf{R})$ of a quadrupole model, when imposing the similar condition of alternating positive and negative charges to the thermal analogue. To validate such a

hypothesis, we take one square unit-structure as a representation under such a source configuration. The displacement vector can be indicated by the relative positions of neighboring cooling and heat sources, whose direction follows the heat flux between the neighboring sources. The temperature distribution is presented in Supplementary Figure 1**c**, and a calculated field directly following $T(\mathbf{R}) = \frac{1}{4\pi\kappa}\frac{1}{|\mathbf{R}|^3}\sum\frac{1}{2}Q_{ij}\widehat{R_i}\widehat{R_j}$ and $Q_{ij} = \int \rho_{heat}\left(3R_iR_j - \|\vec{R}\|\delta_{ij}\right)d^3\mathbf{R}$ is shown in Supplementary Figure 1**d**. These well-overlapped field distributions reveal the significance of such an analog quadrupole model in thermal diffusion.

The key to realizing the quantized bulk quadrupole moment is to host the coexisting positive and negative nearest-neighbor couplings. In order to manifest such quantization and required couplings in heat transport, we integrate tailored advections and thermal coupling strengths to the proposed thermal quadrupole. Here, we design two types of tilted connections to create different thermal coupling orientations (Supplementary Figure 2). These coupling channels connect different terminals of the neighboring sites (advective balls) in *x-z* space, thus enabling the varied heat transfer directions within the channels. We then impose the boundary and advective conditions adopted in the demonstrations (Figs. 2 and 4) to capture the coupling behaviors. To make a fair contrast, we also make parallel channel configurations under different β as references. The temperature distributions of these references (Supplementary Figure 3**a** and **b**) exhibit nearly unbiased isotherms and similar average temperatures in the left and right sites. When we change the channel orientations of the cases enabled by the Hermitian advection (Fig. 2 of the main content), the right sites indicate higher/lower average temperatures and biased deflections of the isotherms (Supplementary Figure 3**b**). The measured locations of the isotherms (the average of imposed heating and cooling sources) further indicate that the changes in channel orientations showcase significance on modifying the temperature distributions within the sites (Supplementary Figure 3**c**). For the cases enabled by the non-Hermitian thermal couplings (Fig. 4 of the main content), similar modes also emerge when modulating the configurations with varied β (Supplementary Figures 3**d** ~ **f**). Based on the above field distributions, the configurations shown in Supplementary Figure 2**a** imply over-coupling behaviors compared with the reference, and the others (Supplementary

Figure 2**b**) give birth to the under-coupling processes. In that case, we could define the effective positive and negative couplings with these over-coupling and under-coupling behaviors.

Supplementary Note 4. Quadrupole topological phases enabled by real- and imaginary-valued bands

4.1 Wannier bands and nested Wannier bands for the proposed two strategies

Since the four-site unit structure proposed in the current work satisfies a quadrupole model, the Wannier bands and nested Wannier bands are the responsible to identify corner states and nontrivial phase. Here, we define the Wannier bands in the Brillouin zone with a Wilson-loop operator along the *x* direction in the parameter space of the heat transport system. The Wilson-loop operator can be indicated as $W_{x,\mathbf{k}_t} = P\exp\left(i \oint A(\mathbf{k}_t)\, dk_{t,x}\right)$ considering the continuity of adjacent units, where $A(\mathbf{k}_t) = i\langle\psi^L_{m,\mathbf{k}_t}|\partial_{k_{t,x}}|\psi^R_{n,\mathbf{k}_t}\rangle$ is the biorthogonal non-Abelian Berry connection for the proposed non-Hermitian heat transport, $|\psi^{R,L}_{m,n,\mathbf{k}_t}\rangle$ are the corresponding right and left Bloch eigenstates (Supplementary Equation 7 ~ 10 of the Supplementary Information) satisfying $\langle\psi^L_{m,\mathbf{k}_t}|\psi^R_{n,\mathbf{k}_t}\rangle = \delta_{mn}$, and *P* is the path-ordering operator. In that case, we have the non-Hermitian Wannier Hamiltonian for the current system $H_W(\mathbf{k}_t) = -\frac{i}{2\pi}\cdot ln\left(W_{x,\mathbf{k}_t}\right)$, whose left and right eigenstates $|v^{R,L}_{x,j,\mathbf{k}_t}\rangle$ are also different under the biorthogonal relation of $\left\langle v^L_{x,j,\mathbf{k}_t}\middle|v^R_{x,j',\mathbf{k}_t}\right\rangle = \delta_{jj'}$ (*j* denotes the Wannier band index). Then, biorthogonal nested Wannier loop along y direction $\widetilde{W}_{x,\mathbf{k}_t}$ can be also defined with the non-Hermitian Wannier-band basis of $w^{R/L}_{x,j,\mathbf{k}_t} = \sum_{m=1}^{N}\left|\psi^{R/L}_{m,\mathbf{k}_t}\right\rangle\left[v^{R/L}_{x,j,\mathbf{k}_t}\right]_m$ under $\left\langle w^L_{x,j,\mathbf{k}_t}\middle|w^R_{x,j',\mathbf{k}_t}\right\rangle = \delta_{jj'}$. Then, the corresponding polarization for the Wannier band sector v_x can be calculated via $p_y^{v_x} = -\frac{i}{4\pi^2}\int dk_{t,x} ln\left(\det\left(\widetilde{W}_{x,\mathbf{k}_t}\right)\right)$. In a similar method, the polarization for the Wannier band sector v_y can be also determined as $p_x^{v_y} = -\frac{i}{4\pi^2}\int dk_{t,y} ln\left(\det\left(\widetilde{W}_{y,\mathbf{k}_t}\right)\right)$. For the quadrupole topological insulator, the Wannier bands should be gapped and symmetric around 0 (v_y and v_x cannot be 0 and $\frac{1}{2}$), and the corresponding polarizations must be nontrivial with a quadrupole invariant $q_{xy} = 2p_x^{v_y}p_y^{v_x} = \frac{1}{2}$, i.e., $p_x^{v_y} = \frac{1}{2}, p_y^{v_x} = \frac{1}{2}$. Here, we calculated the polarizations with the designed parameter spaces of Figs. 2b and 4b of the main content. The Wannier bands and corresponding polarizations shown in Supplementary Figure 4 indicate both the strategies possess gapped Wannier bands and quantized polarizations ($\frac{1}{2}$). Thus, the observed QTI phase, respectively along the real- and

imaginary-valued bands, are nontrivial and topologically protected.

4.2 Gapped real spectrum induced by Hermitian advections

For the quadrupole topological phases induced by Hermitian advection (Fig. 2 of the main content), the same thermal coupling strengths of the intercell and intracell channels ($\beta = 1$) lead to the gapless imaginary-valued band. In that case, the eigenvalues can be expressed as

$$E(\Delta\Omega) = i \cdot 2\left(\frac{\kappa}{\rho c} \cdot \left(k_x^2 + k_y^2\right) + \frac{h}{\rho c a}\right) + \frac{1}{2}(\Omega_I + \Omega_{II})(\cos\theta + \sin\theta)$$
$$\pm \frac{1}{2}\sqrt{\begin{array}{l}\Delta\Omega^2(\cos\theta + \sin\theta)^2 - 8Q_c^2\left(\cos\left(k_{t,x}a\right) + \cos\left(k_{t,y}a\right) + 2\right) \\ \pm 4Q_c\sqrt{2\left(\cos\left(k_{t,y}a\right) + 1\right)\left(8Q_c^2\left(\cos\left(k_{t,x}a\right) + 1\right) - \Delta\Omega^2(\cos\theta + \sin\theta)^2\right)}\end{array}} \tag{11}$$

The changing advection differences $\Delta\Omega$ lead to the hierarchical properties only in the real-valued bands. The eigenfrequencies sorted in these real- and imaginary-valued bands as the function of $\Delta\Omega$ are presented in Supplementary Figures 5**a** and **b**.

The real band structures of the first Brillouin zones under specific advections are presented in Supplementary Figure 6**a**. When the imposed advective magnitudes are same and the directions are opposite ($\Omega_I = -\Omega_{II}$), all the real-valued bands degenerate indicating the gapless structure and the transition between nontrivial and trivial thermal states. When we slightly increase Ω_{II} to $-1.385\Omega_I$ to maintain an advective difference, two gaps emerge between four bands, and the second and third bands are still degenerate. Further increasing Ω_{II} to $-3.154\Omega_I$, all degeneracies lift and a complete open band emerges. In such transitions, we only need to capture the parity inversion between the Γ and X points of the Brillouin zone due to the C_4 symmetry of the square lattice. Here, we consider the lowest band gap (Fig. 1**c**). Parity inversion at the X point is observed as indicated by the flip of the "+" and "-" signs, thus implying the topological phase transition via solely modulating the Hermitian advection represents a class of topological quadrupole phases, embracing the in-gap 0D and gapped 1D topological modes.

4.3 Gapped imaginary spectrum induced by non-Hermitian thermal coupling

For the quadrupole topological phases induced by non-Hermitian thermal coupling (Fig. 4 of the main content), the same advections with quite small magnitudes result in the gapless real-valued band. Then, the eigenvalues can be written as

$$E(\beta)=i\cdot\left(\frac{2\kappa}{\rho c}\cdot\left(k_x^2+k_y^2\right)+\frac{(1+\beta)h}{\rho ca}\right)+\Omega_I\left(\cos\theta+\sin\theta\right)$$
$$\pm\frac{1}{2}\sqrt{\begin{aligned}&-8Q_c^2\left(\cos\left(k_{t,x}a\right)+\cos\left(k_{t,y}a\right)+1+\beta\right)\\&\pm4Q_c\sqrt{\left(2\beta\cos\left(k_{t,y}a\right)+\beta^2+1\right)\left(4Q_c^2\left(2\beta\cos\left(k_{t,x}a\right)+\beta^2+1\right)\right)}\end{aligned}} \tag{12}$$

The varied coupling strengths of the intercell and intracell channels ($\beta\neq 1$) lead to significant hierarchical properties in imaginary-valued bands. The eigenfrequencies sorted in these real- and imaginary-valued bands as the function of β are illustrated in Supplementary Figures 5**c** and **d**. The imaginary band structures of the first Brillouin zones under specific β are shown in Supplementary Figure 6**b**. Based on the C_4 symmetry of the unit-structure, the parity inversion between the Γ and X points of the Brillouin zone of the lowest band gap remarkably showcase the transitions between gapless and gapped phases in imaginary-valued bands. The experimental samples for the demonstrations solely induced by the non-Hermitian thermal couplings are also illustrated in Supplementary Figure 6.

4.4 Numerical quadrupole topological phases of the proposed cases

The numerical temperature distributions and experimental field intensities corresponding to Figs. 2 and 4 are illustrated in Supplementary Figures 7 and 8. Significant features of corner, edge, and bulk states are observed in these validations, which overlap well with the experimental findings in the main contents.

It is noting that the observed behaviors are implemented in fluid transport with a non-vanishing first-order term in the energy equation of the N-S equations (advections). In that case, the temperature fields are available to propagate along the advections (drift-terms) which is non-existing in the omni-directional diffusion of pure conduction. Thus, we can easily capture the behaviors on their temperature distributions. The remarkable differences between their behaviors in temperature fields can be reflected on the irregular and regular distributions (Supplementary Figures 7 and 8), since the convective

temperature fields would also carry the information of the inhomogeneous advective vectors. Considering the imposed velocities via the advective balls, the fluid dynamics (transport) within the entire system (inside and outside the advective balls) are also actuated based on the momentum equation $\rho\frac{\partial \boldsymbol{u}}{\partial t}+\rho(\mathbf{u}\cdot\nabla)\mathbf{u}=-\nabla p+\mu\nabla^2\mathbf{u}+\mathbf{f}$ and exhibit these nontrivial behaviors in the real vector space of pressure and velocity. More specifically for the real-valued band realized by the advections in fluid heat transport, the hierarchical states modulated by advective hermiticity in Fig. 2 can be also exhibited with the distributions of pressures or velocity (Supplementary Figure 9).

4.5 Robustness of the hierarchical states

The eigenvalues of the unit-structure indicate that the critical parameters for enabling the dispersions are the difference of velocities ($\Delta\Omega=\Omega_I-\Omega_{II}$), the basic thermal coupling strength $Q_c=\frac{h}{\rho ca}$ of the adopted fluid, and the ratio of the coupling strengths between intercell and intracell thermal channels (β). These parameters are closely related to the actuated velocities imposed on the hollow advective balls, the working fluid, and the heat exchange areas of the channels. That is, the observed states are robust unless the above parameters are changed. Here, some numerical cases of the fabricated samples shown in Figs. 2 and 4 of the main content are implemented. Taking the corner states as an example, the changes of the materials for coupling channels and advective balls and the initial temperature inputs have little effect on the observations of the QTI phase (Supplementary Figure 10), since the critical parameters for creating the bands are unchanged.

When modulating the above critical parameters, such as the type of working fluid (the product of density and specific heat) and the constructed size of the distance between the centers of the neighboring sites (a), changes occur in the value of the basic thermal coupling strength $Q_c=\frac{h}{\rho ca}$. Such changes further result in the frequency shifts of the boundaries of nontrivial QTI phase, which can be reflected on the changes of fluid or the sizes of coupling channels (Supplementary Figure 11). In that case, the nontrivial states vanish when only modulating these parameters without changing others accordingly. Once modulating these critical values and corresponding parameters simultaneously to satisfy the frequency

shifts, the nontrivial states reoccur again and maintain robustly.

Due to the non-zero nested Wannier bands for these two strategies, the robustness of these hierarchical states is also independent of the site numbers when the critical parameters for creating the bands are unchanged. Additional validations respectively with 192 and 96 sites are implemented (Supplementary Figure 12), whose input parameters are same with the cases in Figs. 2 and 4 of the main content. Significant corner states are observed with the two strategies regardless of the site numbers.

4.6 Robustness of the hierarchical states under defects

We further validate the robustness of these hierarchical states under defects of the lattice. To create the defects, we remove the 16 sites on the lower-right sides of the samples adopted in the main content (Supplementary Figure 6**b**). The other parameters and setups for implementing the two strategies are unchanged (same as the findings shown in Figs. 2 and 4). The numerical temperature field distributions of these cases under defects are presented in Supplementary Figure 13. Significant hierarchical states are also observed by either modulating the Hermitian advections or the non-Hermitian couplings.

The robustness of these corner states shown in Supplementary Figures 13**a** and **d** is enabled by a fractional charge based on the local density of state (LDOS) in Refs. [22, 39] of the main content. In these cases, defects are imposed by removing one entire square-lattice with 16 sites (right-upper insert of Fig. 2a and Fig. 4a) at the lower right corner to create the imperfections in the sample geometry of the system. That is, the C_4 symmetry of the square-lattice remains but the translational symmetry fails within the system under such defects. The sums of local density of state (LDOS) for these cases are presented in Supplementary Figures 14**a** and **b**. Both the findings for the cases induced by Hermitian advections (Supplementary Figures 14**a**) and non-Hermitian thermal couplings (Supplementary Figures 14**b**) indicate that the corners without defects possess half-integer quantized invariant (0.5), which is an important characteristic of the higher-order quadrupole insulator with quantized quadrupole moment based on Wannier bands and nested Wannier bands

(Supplementary Note 4.1). Different from the above regular corner states (without defects) spanning over an angle of $\frac{\pi}{2}$, it also presents that the two neighboring sites of the interior corner, which further generate a trimer, exhibit the fractional charges of 0.25. This is caused by the $\frac{3\pi}{2}$ spanning over the corner with defects, which split the charge of 0.5 into two halves at the neighboring sites. Such fractional charges of 0.25 are also the significant indicator for describing the robustness corner states with fractional charges (Refs. [22, 39]). Such a fractional charge (conserved quantity) is found in the wavefunction of the fluid transport system. Thus, the corresponding states can be also observed in different configurations based on the conservation laws for the fields of energy, mass, and momentum within the fluid, whose transport properties satisfy the wavefunction and further result in the non-zero accumulations and net flow in related fields at the corners. The robust corner states in other field configurations within the fluid (Supplementary Figures 14**c** ~ **f**) further indicate the significance of the conserved quantity (fractional charge).

Supplementary Note 5. Time changing rate of the temperature intensity

The observed behaviors in the main constant are measured at steady states, after the temperature field evolutions reaching equilibrium. Before reaching the observed states, the systems are nonequilibrium. We found that these hierarchical states are also available under time-dependent dynamics, since the temperature field intensities of corresponding sites are always higher than the others at specific times. To characterize such behaviors at nonequilibrium states, we adopt the time changing rate of the field intensity measured by experiments ($\frac{\partial I}{\partial t}, I = \left|\frac{(T^{*}-\bar{T}_{mea})}{\Delta T_{mea}}\right|$ as defined in Figs. 2 and 4 of the main content) (Supplementary Figure 15). The smaller values of $\frac{\partial I}{\partial t}$ imply the small changes in temperature profiles during the nonequilibrium processes, thus revealing the higher intensities at specific sites with energy localizations.

Supplementary Note 6. Potential impacts on general diffusive transports

The realizations of such quadrupole topology in non-Hermitian heat transport indicate a distinct route to modulate thermal behaviors and maintain robustness under changing and complex ambient. Rapidly increasing energy density and heat flux within the thermal techniques in almost all industrial fields, such as conventional energy power and emerging

electronics, raise new demands of higher-efficiency thermal energy utilization, and more intelligent thermal management. Both two aspects directly touch the most fundamental diffusive nature of heat transport, which notoriously restricts the dynamic coherences in principle, let alone the robust heat control and multifarious field reconfigurations. The different and robust profiles of heat transports observed in the work might be a perfect strategy for solving these challenging problems and further pave the way of flexible manipulations. Based on the observed heat patterns, the most intuitive application is heat management, since we can easily localize heat energies at tailored positions of the system independent of the materials and structures. Further exploring the merging states, the localized positions can be anywhere in the system, which is not limited to the bulk, edge, and corners. Such behaviors can be generally realized by creating an internal bound state within the system[1], which break the lattice translational symmetry. As an example, some state-of-art works exploit the butting of topological nontrivial and trivial lattices to form "long-long defects" at their boundaries[1]. Such a method requires the combinations of nontrivial and trivial lattice within the entire system, thus naturally break the translational symmetry at their boundaries. Another potential approach is to insert domain walls at tailored sites (Supplementary Figure 16) in the system possessing one lattice type (such as the square-lattice of the current work). In that case, the neighboring lattices at the interface are mismatched thus giving rise to the internal bound states. Such a property deserves more studies, and it is quite important for exploiting paradigm-shift thermal management in electronic devices and other industrial applications.

It is worth noting that heat transport is one of the typical information carried by fluid dynamics and the extensive transport phenomena, thus we can also reveal similar behaviors in other transport fields under the same framework proposed in this work, and further shed new lights on the unexpected control of mass, charge, momentum, and fluid fields. Such universality of the proposed theory is enabled by the same form of constitutive equations for different transports, such as the Fick low for mass and particle transports, the viscosity for momentum conversion, the drift-diffusion equation for charge carriers, and the Toner-Tu equations for active matter and flocking. An extensive example can be found in Supplementary Figure 9 for the distributions of pressure and velocity of the fluid dynamics for the cases of Fig. 2. For

another extensive representation, we showcase the transports of mass with the two strategies by just changing the boundary conditions to concentrations (Supplementary Figure 17). All these extensive behaviors of other transports imply the generality and importance of the proposed model and the significant impacts on general transport behaviors.

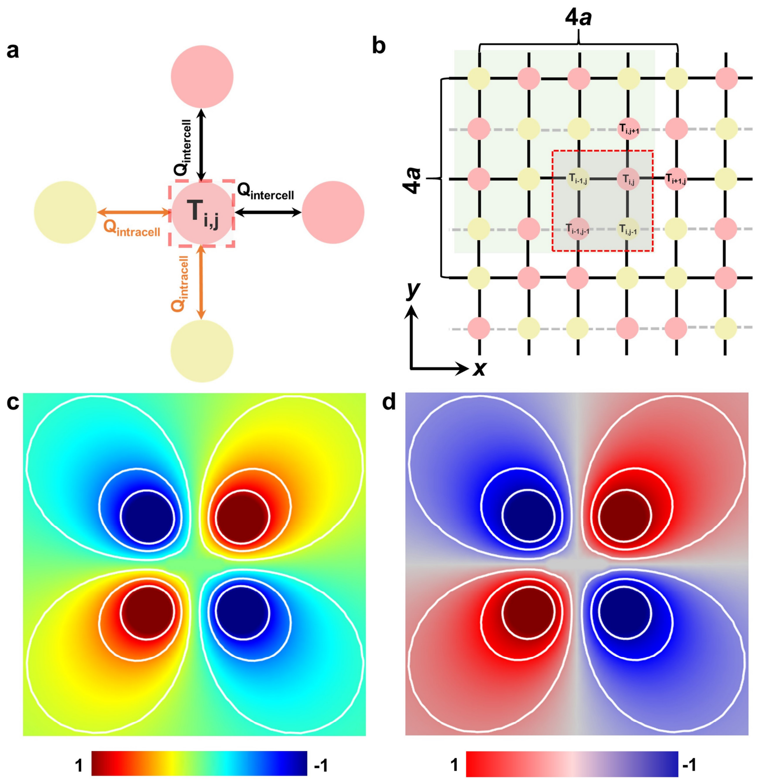

Supplementary Figure 1. General thermal grid system and the thermal analog of a bulk quadrupole. **a**. The heat transport process and corresponding heat exchanges of one site. The orange and black dual-arrow lines denote the intracell and intercell couplings. **b**. The thermal grid system corresponding to a non-Hermitian quadrupole topological insulator. The green shadowed region presents an effective square lattice, and the red border showcases a unit-structure with four sites. **c**. The temperature distribution of a thermal bulk quadrupole in a square unit-structure. **d**. A reference model of a general bulk quadrupole calculated by $T(\mathbf{R}) = \frac{1}{4\pi\kappa}\frac{1}{|\mathbf{R}|^3}\sum\frac{1}{2}Q_{ij}\widehat{R_i}\widehat{R_j}$ and $Q_{ij} = \int \rho_{heat}\left(3R_iR_j - \|\vec{R}\|\delta_{ij}\right)d^3\mathbf{R}$.

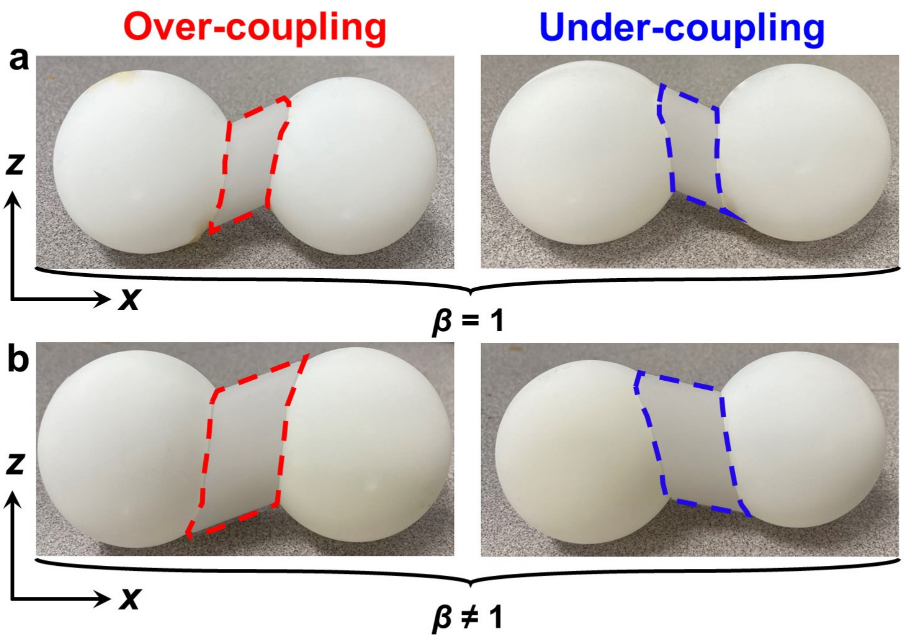

Supplementary Figure 2. Configurations of the over-coupling (red dashed-area) and under-coupling channels (blue dashed-area) of the intracell components. **a**. The intracell channels ($\beta = 1$) for the cases enabled by Hermitian advections (Fig. 2 of the main content). **b**. The intracell channels ($\beta \neq 1$) for the cases enabled by non-Hermitian thermal couplings (Fig. 4 of the main content).

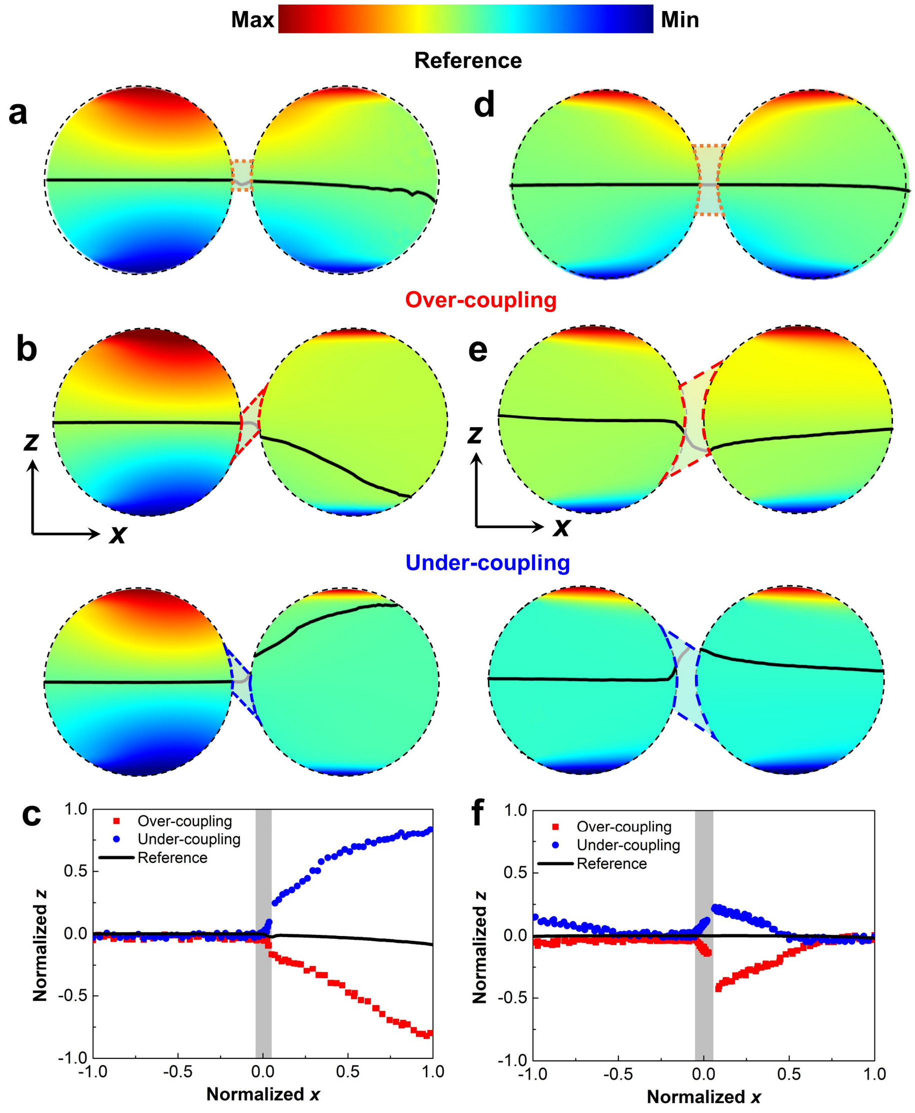

Supplementary Figure 3. Temperature distributions of the intracell channels with tilted connections. The black lines shown in these temperature distributions present the locations of the measured isotherm. **a** ~ **c** illustrate the temperature distributions and measured locations (isotherm) induced by Hermitian advections. **d** ~ **f** present the temperature distributions and measured locations (isotherm) induced by non-Hermitian thermal couplings. The shadow areas in (**c**) and (**f**) indicate the locations of the coupling channels, and the presented data is measured in experiments through thermocouples.

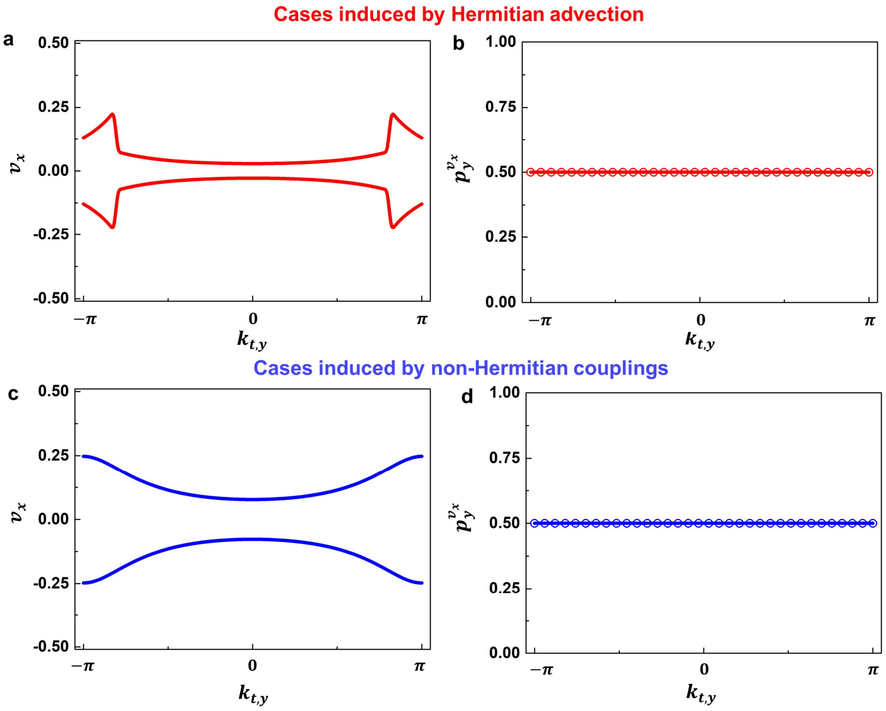

Supplementary Figure 4. The Wannier bands and nested Wannier bands (polarization) for the cases shown in Figs. 2 and 4. **a** and **b** are for the cases induced by Hermitian advections of Fig. 2 of the main content. **c** and **d** are for the cases induced by non-Hermitian couplings of Fig. 4 of the main content.

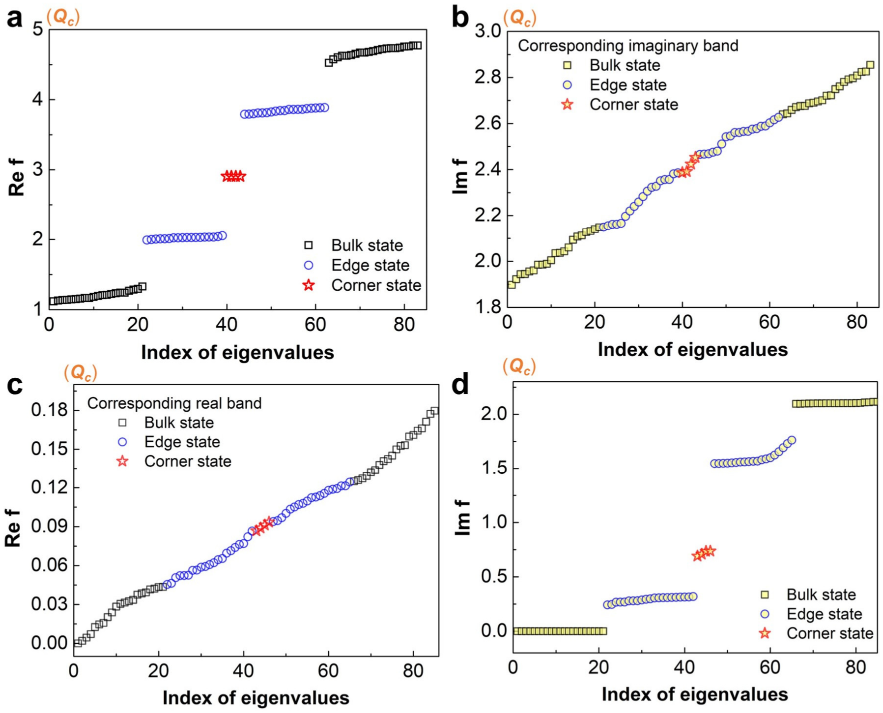

Supplementary Figure 5. The eigenfrequencies sorted in real- and imaginary-valued bands. **a** and **b** illustrate the changing eigenfrequencies based on Supplementary Equation 11. In this case, hierarchical states are only observed in the real-valued band. **c** and **d** indicate the changing eigenfrequencies based on Supplementary Equation 12. It presents hierarchical properties only in the imaginary-valued band, and the real-valued band is gapless.

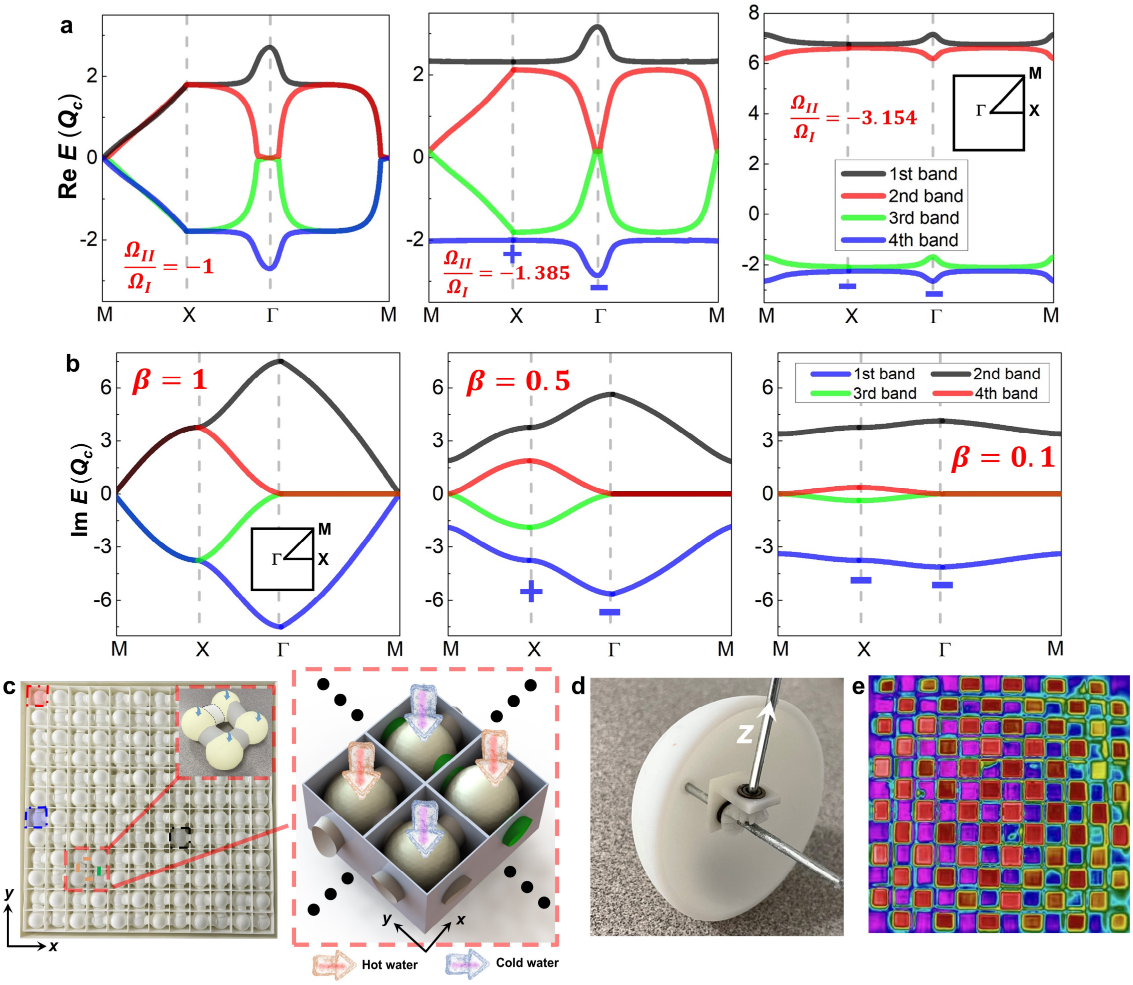

Supplementary Figure 6. Band structures of the first Brillouin zones induced by Hermitian advections and non-Hermitian thermal couplings, fabricated samples in Fig. 4, and the initial thermal profile without advections. **a** denotes the real-valued band structures of the first Brillouin zones under tailored advections. **b** presents the imaginary band structures of the first Brillouin zones under tailored β. The flip of the "+" and "-" signs in (**a**) and (**b**) implies the topological phase transition with the changing advections and β. **c.** Experimental sample for observing the quadrupole topological phases induced by non-Hermitian thermal couplings (Fig. 4 of the main content). The right insert denotes the schematic subgraph of the thermalizing strategy in one four-unit structure with alternative hot and cold waters. **d.** The steering gear set consisting of a pair of bevel gears for modulating the motions of each advective ball. **e.** The initial temperature distribution without advections.

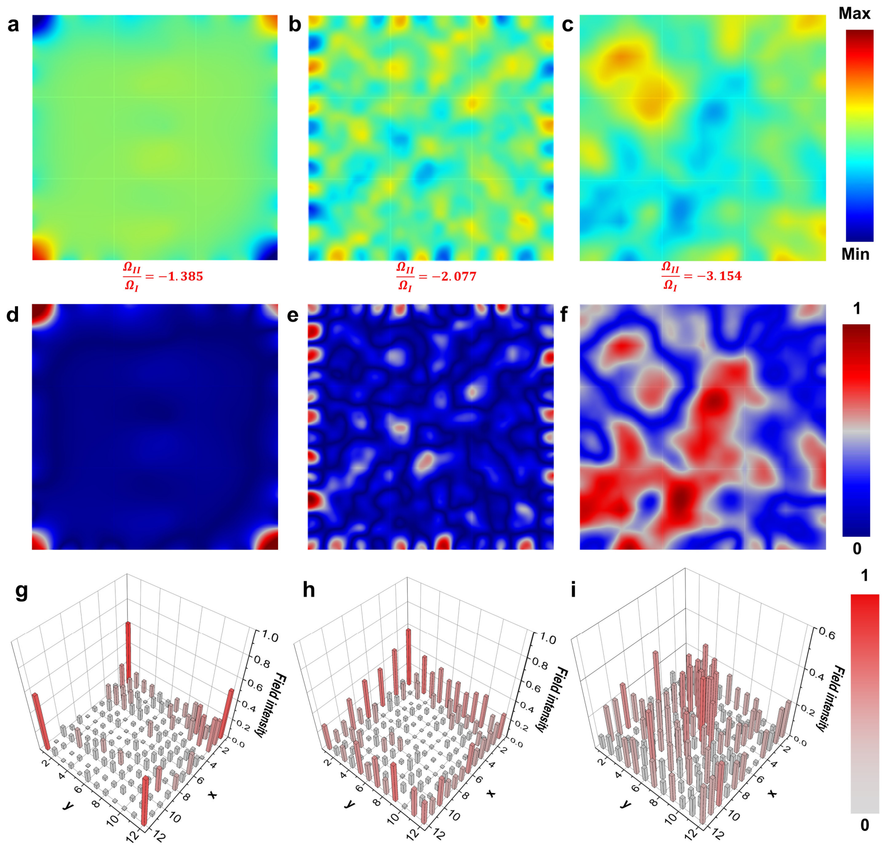

Supplementary Figure 7. Numerical temperature distributions and field intensities of the non-Hermitian quadrupole topological phases solely induced by Hermitian advections (corresponding to Fig. 2 of the main content). **a** ~ **c** present the numerical temperature distributions of the corner, edge, and bulk states. **d** ~ **f** plot the numerical field intensities. **g** ~ **i** illustrate the experimental temperature field intensity profiles corresponding to the cases in Figs. 2**d** ~ **f** of the main content. In the experiments, the field intensities would be larger at the corner and edge as illustrated in **g** and **h**, when adjusting Ω_{II} to the corresponding peaks of edge and corner states. By setting the advections $\Omega_I = 1.3Q_c$ and $\Omega_{II} = -3.154\Omega_I$ (bulk state), the field intensities in the central domain are much higher than the ones at the corners and edges (Fig. **i**).

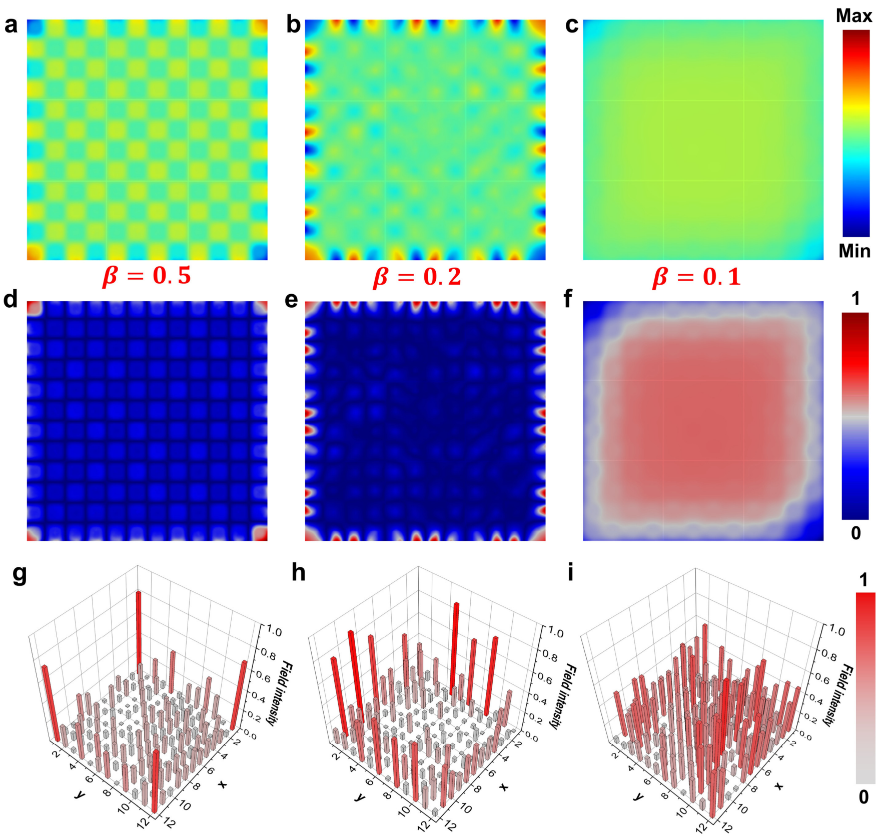

Supplementary Figure 8. Numerical temperature distributions and field intensities of the non-Hermitian quadrupole topological phases solely induced by non-Hermitian thermal couplings (corresponding to Fig. 4 of the main content). **a** ~ **c** present the numerical temperature distributions of the corner, edge, and bulk states. **d** ~ **f** plot the numerical field intensities. **g** ~ **i** illustrate the experimental temperature field intensity profiles corresponding to the cases in Figs. 4**d** ~ **f** of the main content. In the experiments, the field intensities would be larger at the corner and edge as illustrated in **g** and **h**, when adjusting β to the corresponding peaks of corner ($\beta = 0.5$) and edge ($\beta = 0.2$) states. By setting $\beta = 0.1$ (bulk state), the field intensities in the central domain are much higher than the ones at the corners and edges.

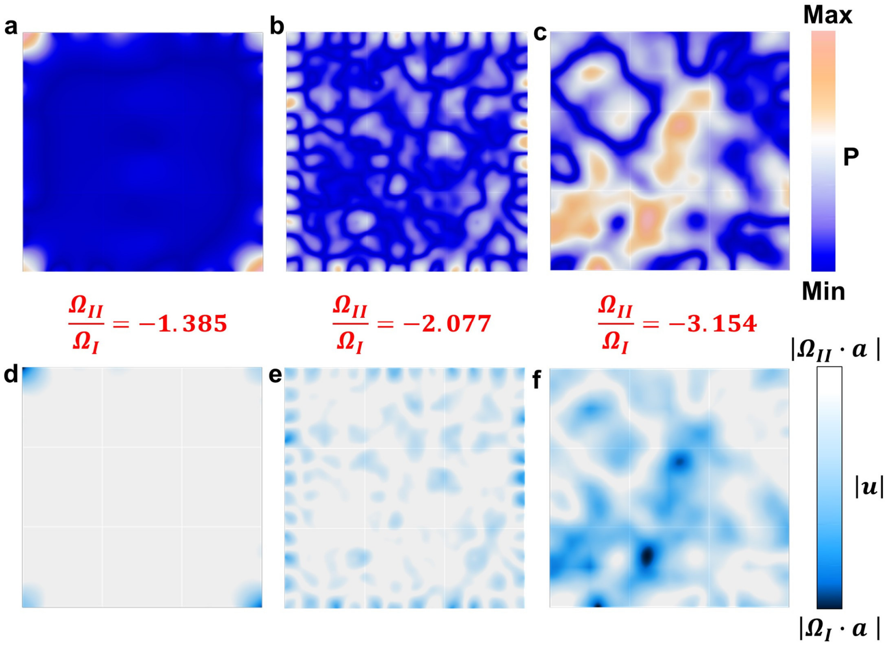

Supplementary Figure 9. Pressure and velocity distributions of the fluid dynamics for the cases induced by Hermitian advections. **a** ~ **c** are the pressure distributions of the corner, edge and bulk states, and **d** ~ **f** are the velocity distributions of the corner, edge and bulk states.

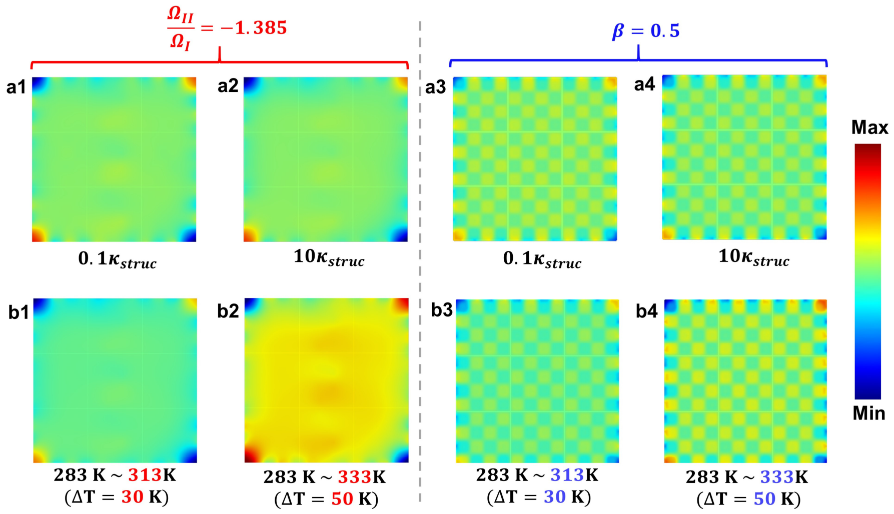

Supplementary Figure 10. Corner states observed in the temperature distributions under different structural materials and initial temperature intervals. **a1** ~ **a4** present the temperature distributions of the corner states under different materials of the structure elements. We take two different conductivities of the structural materials to validate the robustness for these cases ($0.1\kappa_{\text{struc}}$ and $10\kappa_{\text{struc}}$, κ_{struc} denotes the one adopted in the main content). Among them, **a1** and **a2** are the cases induced by Hermitian advections, and **a3** and **a4** are the cases induced by Hermitian couplings. **b1** ~ **b4** exhibit the corner states under different initial temperature intervals. Two different initial temperature intervals ([283 K ~ 313 K] and [283 K ~ 333K]) from the one of main content ([283 K ~ 323K]) are adopted here. Among them, **b1** and **b2** are the cases induced by Hermitian advections, and **b3** and **b4** are the cases induced by Hermitian couplings.

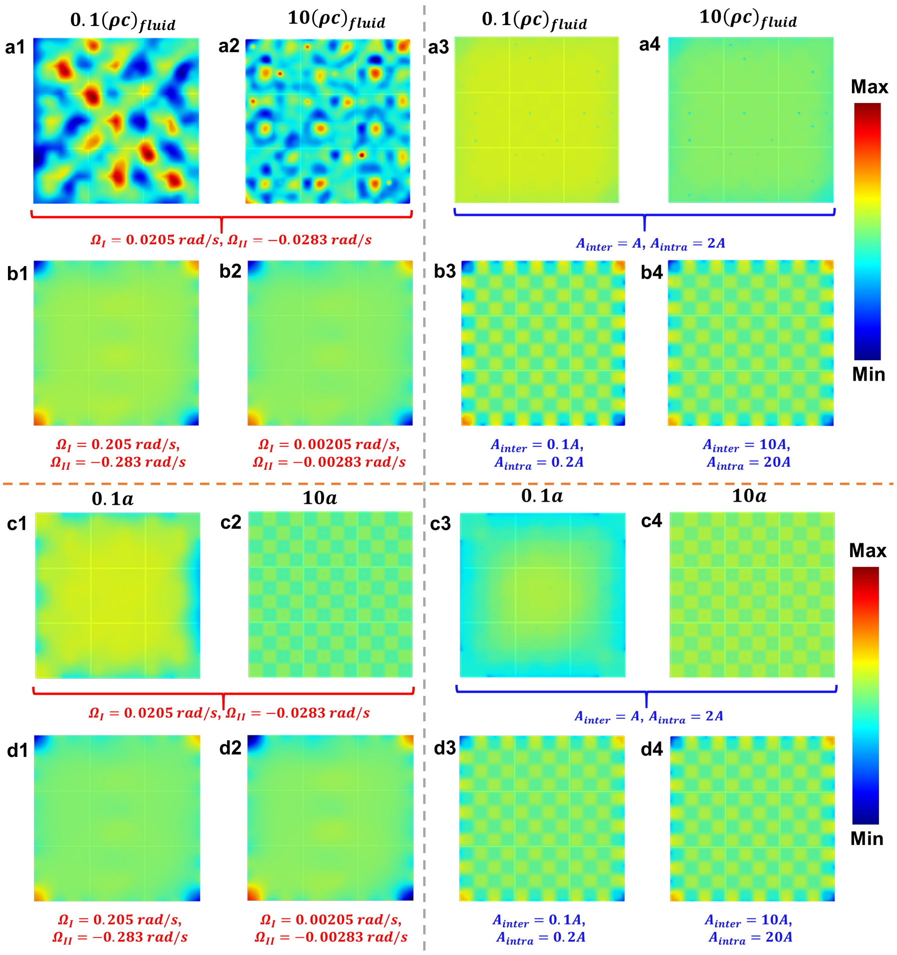

Supplementary Figure 11. Corner states under different fluids and constructed sizes. Columns **a** and **b** respectively denote the temperature distributions of the vanishing and robust corner states via adopting different fluids. Among them, column **a** presents the vanishing corner states in the temperature profiles by only adjusting the products of fluid density and specific heat to $0.1(\rho c)_{\mathrm{struc}}$ and $10(\rho c)_{\mathrm{struc}}$ (the term of $(\rho c)_{\mathrm{struc}}$ is the one adopted in the main content) without other parameter changes. Specifically, **a1** and **a2** are the cases induced by Hermitian advections**,** and **a3** and **a4** are the cases induced by non-Hermitian couplings. Column **b** denotes the robust corner states by simultaneously adjusting the products of fluid density and specific heat and related parameters corresponding to the two strategies. Specifically, **b1** and **b2** are the cases induced by Hermitian advections with satisfied velocities**,** and **b3** and **b4** are the cases induced by non-Hermitian couplings with satisfied heat exchange areas for the channels (A is the heat exchange area for intercell channels used in the main content). Columns **c** and **d** respectively exhibit the temperature distributions of the vanishing and robust corner states via adopting different constructed sizes. Among them, column **c** presents the vanishing corner states by only adjusting the

distance between the centers of the neighboring sites to 0.1*a* and 10 *a* (*a* is the one adopted in the main content) without other parameter changes. Specifically, **c1** and **c2 a**re the cases induced by Hermitian advections**,** and **c3** and **c4** are the cases induced by non-Hermitian couplings. Column **d** presents the robust corner states by simultaneously adjusting distance between the centers of the neighboring sites and related parameters corresponding to the two strategies. Specifically, **d1** and **d2** are the cases induced by Hermitian advections with satisfied velocities, and **d3** and **d4** are the cases induced by non-Hermitian couplings with satisfied heat exchange areas for the channels.

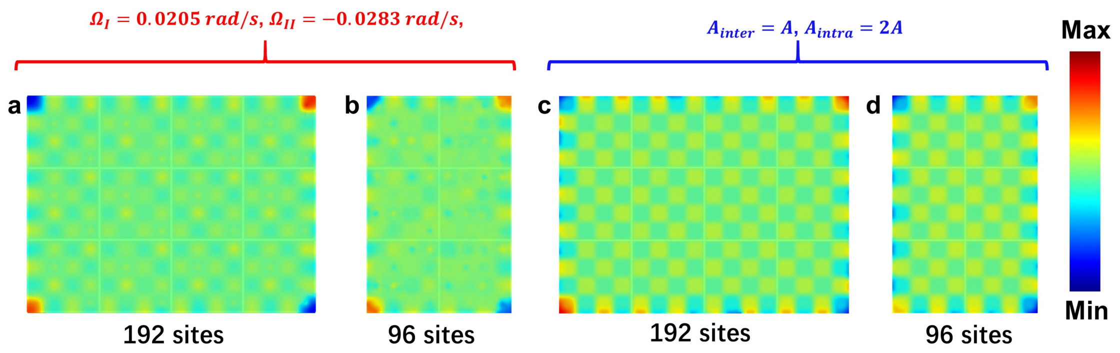

Supplementary Figure 12. Corner states under some different site numbers. **a** and **b** present the robust corner states induced by Hermitian advections, which respectively possess 192 and 96 sites without other parameter changes. **c** and **d** indicate the robust corner states induced by non-Hermitian couplings, which respectively possess 192 and 96 sites without other parameter changes.

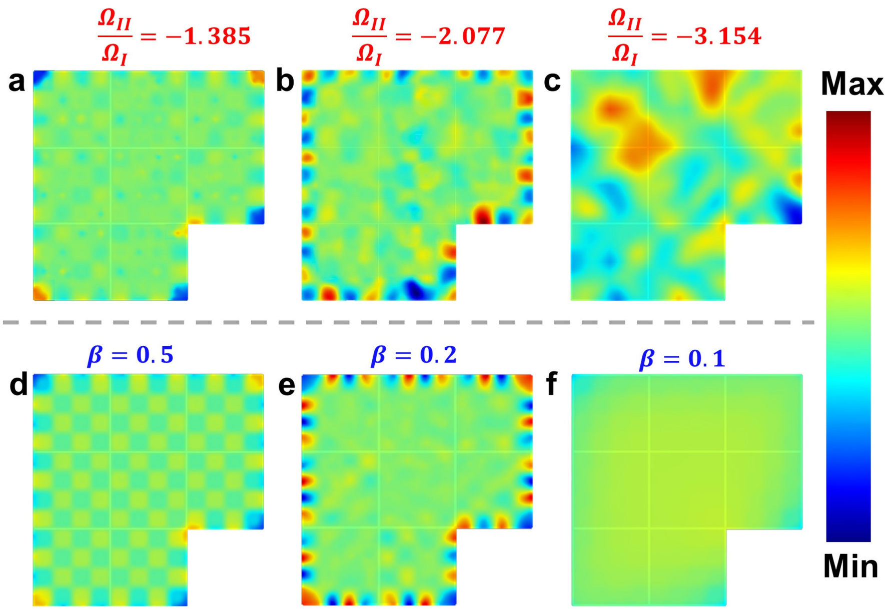

Supplementary Figure 13. The robustness of hierarchical topological states under some defects of the lattice. **a** ~ **c** are the corner, edge, and bulk states induced by Hermitian advections in real-valued bands. **d** and **e** are the hierarchical states induced by non-Hermitian couplings in imaginary-valued bands.

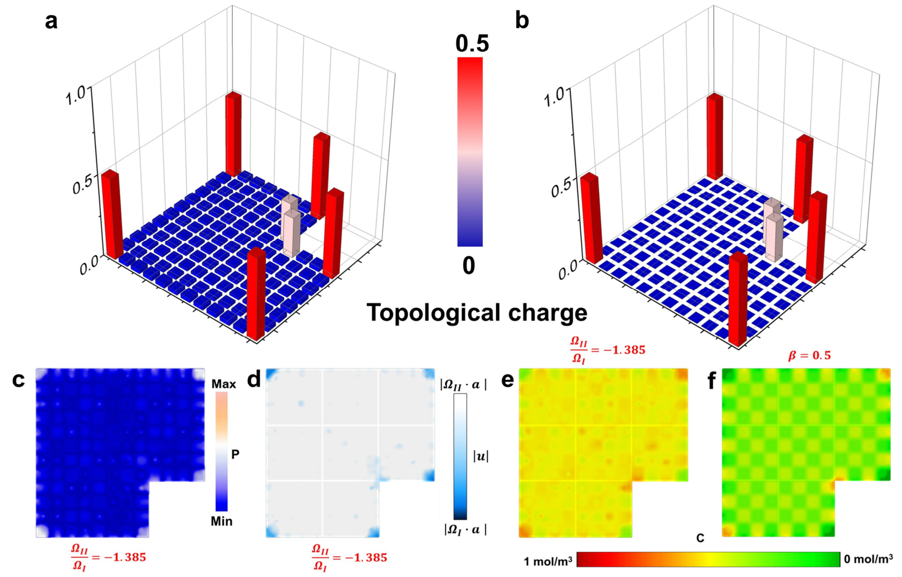

Supplementary Figure 14. Topological charge and corner states for different fields under the defects of imperfections in the sample geometry. **a** and **b** respectively denote the topological charges (quantized quadrupole invariant) for the cases induced by Hermitian advection and non-Hermitian couplings. Both these cases showcase fractional charges of 0.5 at the regular corner without defects and 0.25 at the neighboring sites of the interior corner at the sample defects. **c** ~ **e** present the momentum fields (pressure and velocity) and mass concentration field for the cases induced by Hermitian advections (the imposed advections are same with the cases shown in Fig. 2a of the main content). **f** plots the mass concentration field induced by non-Hermitian coupling (β = 0.5) under near-zero and same advections (quasi-conduction system). All these field distributions indicate significant corner states protected by these topological charges.

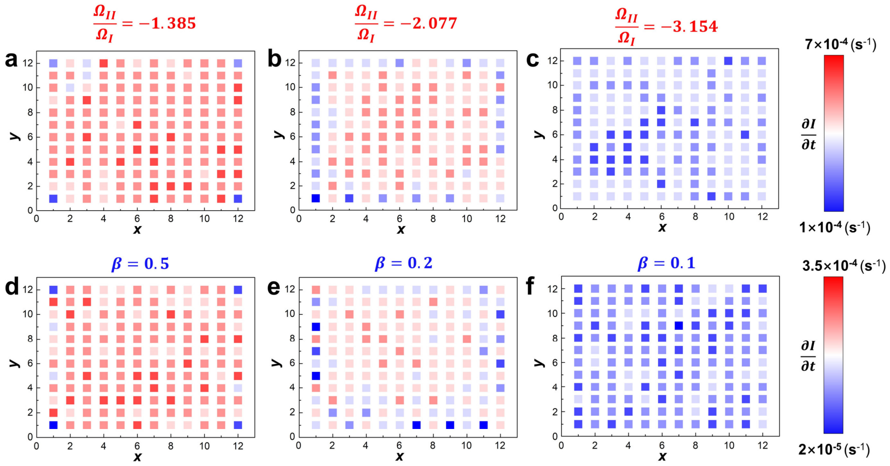

Supplementary Figure 15. Time changing rate of the experimental field intensity of each site. **a** ~ **c** respectively denote the time changing field intensities for each site of the corner, edge, and bulk states induced by Hermitian advections. **d** ~ **f** present the time changing field intensities for each site of the corner, edge, and bulk states induced by non-Hermitian couplings.

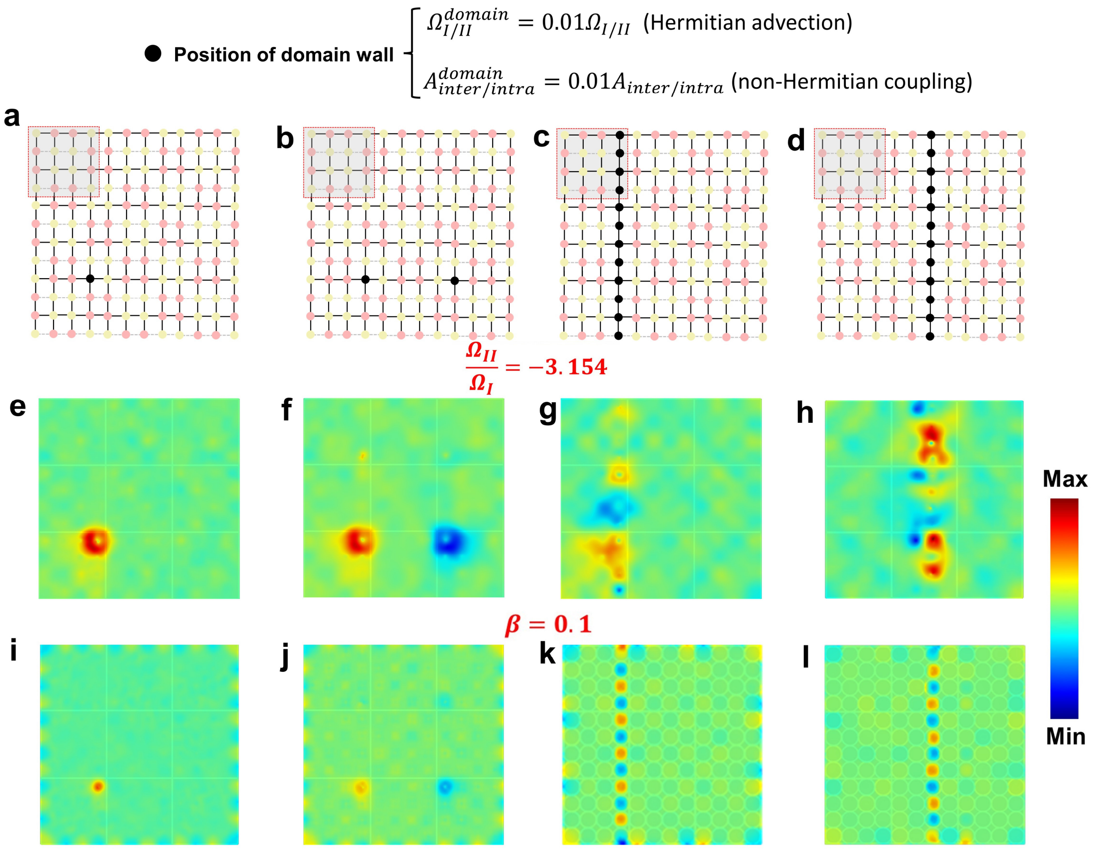

Supplementary Figure 16. Bound states in arbitrary positions of the system with domain walls. **a** ~ **d** denote the sites where domain walls are set within the system (black dots). In these illustrations, we maintain the advections and couplings for the bulk states shown in Fig. 2f and 4f of the main content in corresponding cases. Then, the related domain walls can be created by reducing the on-site advections (coupling channel areas) to 0.01 time to the original one for the case induced by Hermitian advections (non-Hermitian couplings). **e** ~ **h** plot the temperature fields of the internal bound states (inner-corner and inner-edge states) with different quantities and positions induced by the Hermitian advections. **i** ~ **l** present the temperature fields of the corresponding internal bound states induced by the non-Hermitian couplings.

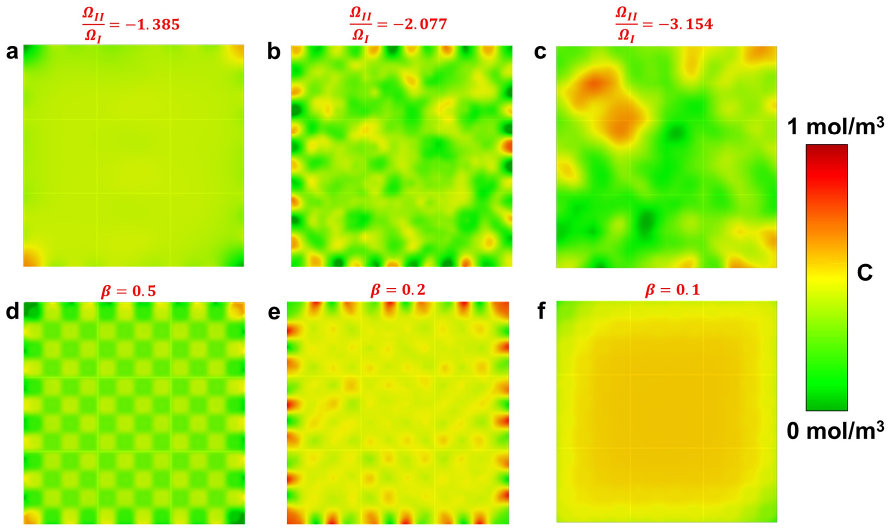

Supplementary Figure 17. Extensive impacts on the transports of mass within fluid. The configurations of Hermitian advections and non-Hermitian couplings are unchanged. **a** ~ **f** respectively exhibit the hierarchical states in mass concentrations by replacing the initial boundary conditions to 1 mol/m^3 and 0 mol/m^3. Among them, **a** ~ **c** are induced by Hermitian advections, and **d** ~ **f** are induced by non-Hermitian couplings. The configurations of advections and coupling ratios are same with the ones of Figs. 2 and 4.

Supplementary References

1. Takata, K., Notomi, M. Photonic topological insulating phase induced solely by gain and loss. *Phys. Rev. Lett.* **121**, 213902, (2018).
